# Efforts of a Kansas Foundation to Increase Physical Activity and Improve Health by Funding Community Trails, 2012

**DOI:** 10.5888/pcd11.140356

**Published:** 2014-11-26

**Authors:** Katie M. Heinrich, Joseph Lightner, Katherine B. Oestman, S. Morgan Hughey, Andrew T. Kaczynski

**Affiliations:** Author Affiliations: Katie M. Heinrich, Joseph Lightner, Kansas State University, Manhattan, Kansas; Katherine B. Oestman, Riley County Health Department, Manhattan, Kansas; Andrew T. Kaczynski, University of South Carolina, Columbia, South Carolina.

## Abstract

**Introduction:**

Trails are associated with increased physical activity; however, little is known about the process of building trails by various types of organizations. From 2005 through 2012 the Sunflower Foundation: Health Care for Kansans (Sunflower) funded multiple organizations to construct 70 trails of varying lengths and surfaces in municipalities, schools, and communities across Kansas. The purpose of this study was to assess the process of developing and implementing community trail projects across Kansas with funding from a public foundation.

**Methods:**

In 2012, we stratified funded organizations by type and conducted proportional random sampling to select 20 key informants from those organizations to participate in structured telephone interviews. Interviews were recorded and transcribed verbatim. Two researchers coded interview transcripts according to issues identified by participants.

**Results:**

Issues associated with trail-building identified as important were collaboration among groups, unexpected construction costs, champions for the project, and level of difficulty of construction. Participants indicated that trails facilitated physical activity. Trails were integrated into communities through events such as walking events and other promotional efforts; these efforts were thought to increase trail use. The perceived outcomes of building the trails included providing the community with a physical activity resource, inspiring the community to start additional trail projects, and increasing the physical activity of local residents.

**Conclusion:**

Sunflower’s funding was instrumental in developing trail projects to provide new physical activity resources across Kansas. Public health practitioners seeking to increase physical activity should seek funding from foundations that focus on health.

## Introduction

Physical activity is essential to maintaining health, particularly for preventing obesity, diabetes mellitus, cardiovascular disease, and several forms of cancer (1). Despite the associated health benefits, most Americans do not meet established physical activity recommendations (2,3). Increasing physical activity levels across populations requires multilevel and innovative interventions, including making choices for transportation and recreation through physical activity both available and appealing (4). The environments in which we live, work, and play influence our health; specifically, the built environment influences decisions regarding physical activity for transportation and recreation (5,6). Hiking trails are one component of the built environment that promotes physical activity (7–9).

Previous trail-related studies focused on identifying both demographic and environmental correlates (ie, barriers to and facilitators of trail use) (9). New community walking or biking trails are often associated with increased physical activity levels (10–14); for example, one study demonstrated that parks with trails were more likely to be used for physical activity than parks without trails (15), and another study showed that a new walking or biking trail constructed to connect homes to destinations such as parks or shopping areas significantly increased recreational walking or biking trips (16). In addition, trail users are more likely to meet physical activity recommendations than nontrail users (17). Studies used experimental designs (eg, natural experiments) to examine the impact of trails on physical activity levels among community members; one study found significant increases in total physical activity (16), and another found no changes (18).

Brownson et al described how community organizations and an academic team worked collaboratively to develop and promote trails in rural areas and the impact the trails had on physical activity levels of community residents (19). They found that local public and private agencies were usually willing to donate both time and materials for trail construction and maintenance (19) but noted that more research was needed to identify effective ways to promote trail use (19).

Trail investments can be cost effective. An examination of construction and maintenance costs for 5 trails in Lincoln, Nebraska, found that every dollar invested led to $2.94 in direct medical benefit (the cost-benefit ratio was calculated by dividing the direct medical cost saving by the total trail costs); cost per use was $0.27 to $0.78, substantially less than a health club membership (20). Trails can also increase property values and resulting tax revenues (21). However, policy barriers (eg, local budget cuts, prohibitive design standards) can hinder trail completion as can conflicts between invested entities (ie, funding organizations, government representatives, advocacy and community groups, engineers, and local businesses) (21). An examination of trails constructed through federal, state, or local funding found that trail-building required committed people, successful collaborative partnerships, a trail champion, perseverance, and involvement of the community (21).

Government organizations, public health researchers and practitioners, and private and public foundations have all played a major role in improving public health (22). Many foundations adopted funding initiatives and policy statements that reflect major public health needs and thus are a frequently sought source of funding for projects that further their mission (23). Foundations often fund infrastructure projects, including trail construction, that would otherwise not be funded.

Despite research showing that trails are a good financial investment and are linked to greater physical activity, few studies examined the process of trail development funded by a foundation with the participation and involvement of multiple organizations. The purpose of this study was to assess the process of developing and constructing community trails funded by the Sunflower Foundation: Health Care for Kansans (Sunflower) as well as key factors in the trail-building projects that helped advance the foundation’s goal of improving the health of Kansans.

## Methods

### Study design and participants

Sunflower was established in 2000 as a statewide public foundation and grant-making organization with the mission of helping people and communities across Kansas achieve and maintain optimal health (24). The Sunflower Trails program was established in 2005 to support trail-building as a means of increasing opportunities for outdoor physical activity statewide. This program provided funding for the infrastructure necessary for building, expanding, or improving trails in Kansas.

From 2005 through 2012, Sunflower funded 70 trail construction projects for 40 municipalities, 15 schools, and 15 communities. Depending on the resources of each community, trails varied in length (0.20 to 8.8 miles) and surface material (eg, crushed rock, asphalt). Sunflower contracted with university researchers in 2012 to conduct a qualitative evaluation study of the Sunflower Trails program. To capture factors unique to each setting, trail projects were stratified by type of organization funded (ie, municipality, school, community). Proportional random sampling was used to select 1 key informant from 20 of the 70 projects to participate in telephone interviews. Data from the interviews were used to inform a second phase of the evaluation, where key contacts from all 70 trail projects were asked to complete an online survey. However, this article focuses solely on the interview phase of the project. The study was approved by Kansas State University’s institutional review board. All participants gave oral informed consent.

### Data collection

Sunflower provided a list of primary contacts in funded organizations for each trail project. The 20 randomly selected key informants were called to schedule an interview. Participants who could not be reached by telephone were contacted by e-mail. One person contacted did not respond, so another was randomly selected from the same organization type to replace that informant, for an overall response rate of 100%. Structured interviews were conducted with each selected key informant in 2012. The interview consisted of 12 open-ended questions that were developed jointly by the researchers and a Sunflower staff member responsible for oversight of the trail program. In developing the interview questions, we took into consideration the facilitators and barriers identified by previous researchers (19,21). Interview questions were organized into 3 major categories following the timeline for each project: collaborative process of building the trails, integration of the trails within communities, and impact of the trails on physical activity. For the collaborative process, key informants were asked how they learned about Sunflower’s grant funding; how they worked on preparing their application, including whom they worked with and whether there was a trail champion; how the various involved organizations worked collaboratively to build the trail; how they would assess the difficulty of constructing the trail; and how they handled any extra costs needed for the trail. For trail integration in the community, key informants were asked how plans for the trail were announced to the public and how the completed trail was marketed or advertised; who was responsible for maintaining the trail after construction; and were there any events, promotions or programs associated with the trail. For trail impact, key informants were asked about specific groups of users and how they thought the trail had changed physical activity levels; were there any future plans for the trail; and had building the trail led to other trail-related projects. A complete interview guide is available in the Appendix. The structured telephone interviews lasted an average of 25 minutes.

### Analysis

All interviews were audio-recorded and transcribed verbatim, and transcripts were verified by participants. Two research assistants independently reviewed all 20 interview transcripts and developed a master list of repeated ideas generated from interviews (25). Research assistants discussed the ideas and collaboratively organized them into categories. Interview transcripts were then coded by each research assistant, and transcripts were compared for agreement. Any discrepancies were resolved through discussion. Finally, the research assistants collaborated on coding themes in order to identify overarching themes.

## Results

Ten overarching themes were identified in the interview responses (Table). The themes were grouped by the interview framework into collaborative processes of building the trails, integration of the trails within communities, and impact of the trails (Figure). 

**Table:** Themes and Sample Comments From Interviews With Key Informants (n = 20) from the Trail Project (N = 70), Kansas, 2012

Category/Themes	Example Comments
**Collaborative process of building trails**	
Collaboration between groups	“The collaborative process included representatives from Thrive County staff [name omitted], city staff including . . . the public works director . . . and also the parks committee.” “The school district did all of the work and put it all together. So it was me working with people from the service center developing the trail landscape, but it was all public school employees that did it.”
Additional costs for trail construction	“There were a few extra costs that the local contractor absorbed . . . considered it a donation.” “No, there were no extra costs because it was a competitive bid.”
Champions for the trails	“It was one of my physical education teachers.” “I’d definitely say . . . the coordinator for our . . . coalition. In addition, maybe just a couple of local individuals.”
Level of difficulty to construct the trails	“In my mind, I was thinking, “Oh let’s just slap a trail out there. How hard can it be?” It’s pretty hard. A lot of people have to sign off on those things. It took basically a whole year to make that process happen.” “It actually went rather smooth because of all the support we had with the community.”
**Integration of the trails within communities**	
Events held at the trails	“Our PTO sponsors a walk-a-thon as one of their fundraisers.” “Just this past weekend the Humane Society had a pet expo going on and they had a pet jog/walk around the park, so they naturally used that trail as well.”
Promotional efforts for the trails	“We had a ceremony, a huge ceremony where we actually had people from the Sunflower Foundation there and the superintendent of schools, we had a lot of dignitaries there and a big, big party to celebrate the opening of the trail.” “It was announced in the paper and then we did have, we talked about it on a local radio show.”
Maintenance of the trails	“The county . . . they come about every 6 weeks just to make sure there is nothing on the trail.” “The building principal is the one that would be ultimately responsible.”
**Impacts of the trails**	
Serving as a key physical activity community resource	“I noticed there are a lot more people out walking because they have a controlled area that is well lit and actually gives them a mark of how far they’re walking.” “You know, parents will come over to bring their kids to play on the playground and go walk themselves. It’s of the size you can do that and reasonably keep an eye on your kid and create a family activity.”
Additional or future trail-related projects	“We hope to have plans in the future to add to some things in that area where that park is, some benches, some trees.” “We would like to extend it from the end of it to the city limits.”
Assessments of trail use	“Well actually we did, at the beginning, put in a trail counter so we keep track every 3 months on how often the trail is used . . . from July 7 through October 19, 2011, . . . we had over 10,000 total counts on our trail counter.” “We have not and I know we need to do that.”

**Figure:**
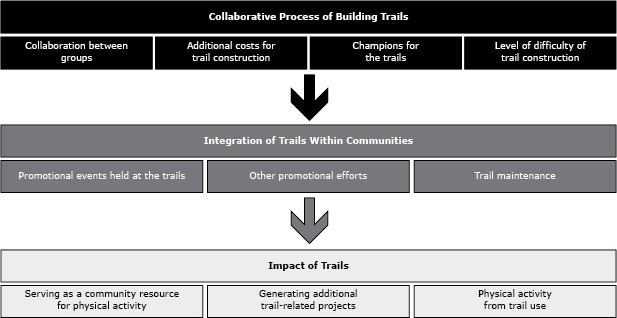
Key themes that emerged from interviews about the process of developing and implementing community trail projects, Kansas, 2012.

### Collaborative process of building trails

“Collaboration among groups” was the theme that emerged most frequently from interviews. Key collaborators identified included local businesses (eg, grocery stores, banks), city administrations and departments (eg, public works, parks and recreation), grass roots and nonprofit groups, community organizations (eg, Lions Club, Boy Scouts), local school districts, health and wellness organizations (eg, hospitals, Red Cross, health coalitions), county government (eg, commissioners, extension offices), community members, local convention and tourism groups, universities, land developer and homeowner associations, health departments, Safe Routes To School (http://www.saferoutesinfo.org/) projects, and state government (eg, transportation departments). Collaborations were used to develop project ideas, obtain building permits and site or property information for rights-of-way, to work with adjacent property owners, to write grants, and to plan and design trails. City street crews or construction companies constructed most trails, and local electric companies installed lighting. Some trails were constructed entirely by volunteers, including residents, Boy Scouts, college students, and members of community service organizations. School trails often involved collaborations among parents, school boards, teachers, and neighborhood associations. A key aspect of the successful collaborations was joint decision-making by all parties, often involving discussions or town-hall meetings.

One criterion for receiving funding from Sunflower was that the applicant provide matching funds. Some participants described how any additional costs for trail construction were either covered by the city, generously absorbed by local businesses, covered through fundraisers, or even paid by community residents, whereas others said there were no additional costs beyond those covered by Sunflower’s funds and the matching funds. Most participants described “champions for the trails” as city staff members or departments, community members or organizations, school staff, elected officials, and local coalitions.

Almost one third of participants reported that constructing the trail was more difficult than expected, although they were able to work collaboratively to address the challenges. For example, because of stakeholder feedback, 1 trail was redesigned several times. Another participant reported costs exceeding expectations and problems convincing board members that the trail would be used by community members. Another reported delays resulting from dealing with property owners. Most participants reported that the trail was as easy to complete as expected, often reporting that they had completed similar construction previously.

### Integration of trails within communities

Several key informants described conducting events at the trails to help integrate trails into the community (eg, mountain bike races, 5K runs, “haunted trails,” walkathons, educational programs, fitness challenges, and fundraisers). These events were successful in promoting trail awareness by attracting both community residents and visitors. Additional promotional efforts were mentioned, such as news and website advertisements, ceremonies, flyers, brochures, and events such as walkathons and 5Ks.

Various groups assumed responsibility for trail maintenance, including city government departments, a sponsoring organization (eg, YMCA, hospital, school), county government, or occasionally volunteers (eg, residents, trail organizations). Some trails were designed to require minimal maintenance through the use of natural materials, whereas the design of others required periodic resurfacing.

### Impact of the trails

Another prominent theme that emerged in participant responses was related to trails serving as a key physical activity resource in the community. Many trails were built in rural communities with few physical activity resources. These communities used the trails to connect or add to their parks and playgrounds. Trails also provided access to utilitarian destinations (eg, grocery store, work) for walkers and cyclists. Participants perceived that trails increased physical activity levels for a variety of users (eg, children, exercisers, the elderly, families), served multiple purposes (eg, workplace exercise breaks, access to fishing, school recess, summer school programs, physical education classes), and was a safe resource for outdoor activities (eg, lighted area, soft surface, vehicle-free area). For some rural communities, trails fostered social interaction and engagement among community members who used them for physical activity.

The third theme, “additional or future trail-related projects,” included establishing or having plans for trail improvements, additions, or extensions; health and wellness activities; and bike or pedestrian task forces (ie, grass-roots citizen groups who advocated for trail construction and use). Several participants reported applying for and obtaining additional grants for other trail-related projects, Safe Routes to School initiatives, or health promotion projects. Some planned to develop a more complete trail network.

Few participants had conducted an assessment of trail use. A few had conducted informal assessments such as installing trail counters or creating exercise program logs. These efforts provided data indicating the trails were being used for physical activity by people in the community.

## Discussion

Walking and cycling trails are important components of the built environment, and their effect on physical activity, obesity, and health is increasingly of interest to researchers and professionals in public health, parks and recreation, transportation planning, and other fields (8,9,16–18). Despite a growing demand for trail research and construction in communities, few studies have focused on important process-related elements of trail development or the in-depth perceptions of diverse stakeholders involved in the development process (19). This study examined the unique efforts of a state foundation to develop and construct community trails, and it provides valuable data on issues related to the collaborative process of trail development and construction, integration of trails within communities, and perceived effects of trails. As such, this study can guide researchers to a better understanding of the reasons underlying use or nonuse of trails by residents, and it can be used for public health and planning scholars and practitioners interested in fostering and studying successful community trail projects.

All projects involved collaborations, often across multiple disciplines and organization types throughout the trail-development and construction process. Although these collaborations to change the built environment required significant commitments of time, support, and resources (13), collaborators felt the trails served as important resources that promoted physical activity and fostered social relationships around the built environment’s features (26). Many trails were built in rural communities with few physical activity resources. For example, the Wyoming Valley Wellness Trails Partnership helped link rural, urban, and suburban communities by constructing walking and biking trails and promoting opportunities for physical activity (27). Perceptions of the effectiveness of trail development align with previous findings suggesting that the presence of built environment features (eg, parks, trails, sidewalks) positively influences the physical activity levels of residents who live near these features (10–14).

Similar to results of Eyler et al, this process evaluation found that collaborations among various types of organizations were necessary to complete trail projects from beginning to end: idea development, grant writing, planning and design, construction, promotion, and use by community members (21). Interorganizational collaboration facilitates complex problem-solving by using specific skills from various organizations to complete tasks that could not be accomplished by 1 organization (28,29). For example, partnerships between city administrations and parks and recreation departments facilitated easier construction of paved trails because of existing capacity and resources in the city organization. These partnerships helped address some of the policy barriers identified by Eyler et al (21). Some trail projects relied more heavily on the physical efforts of community organizations, residents, and volunteers, which often resulted in construction of natural-surface trails. Communication among groups was important and facilitated greater stakeholder and community support for the trails (28,29). This study also was able to identify methods of promoting trail use, such as 5K events, advertisements, and ceremonies, which helped integrate the trails into their communities (21).

Our study has limitations. Although we randomly selected key informants from the 70 trail projects, we probably missed obtaining valuable perceptions from team members in the other 50 projects. Our study’s sample of key informants and their trail-building projects, the organizations involved in those projects, and the local foundation that funded them are unique to Kansas; therefore, results may not be applicable to other settings. However, we aimed to select key representatives from a variety of organizations to get the most accurate representation possible of the trails project. Future studies should determine each key informant’s specific role on the trail projects and include key informants from the funding agency, collaborating organizations, and community residents. The structured interviews lasted on average about 25 minutes; a more in-depth interview may have provided more detailed responses to the interview questions. Additionally, the results reported by the key informants regarding physical activity reflect only the views of those informants and do not capture objectively measured physical activity levels of community members. The study’s qualitative methods captured an in-depth view of the process of trail development and construction in 20 communities in Kansas, which can inform similar efforts in other communities and states.

Evaluation results clearly indicated that the trail funding provided by Sunflower helped fill a need for Kansas communities by creating trails to serve as a physical activity resource and by stimulating ideas for further improvements and projects. Although little evaluation was conducted to capture the actual effect of the trails on the physical activity levels of users, the completed trails have the potential to improve the health of Kansas residents. Public health practitioners seeking to influence physical activity should look for foundation funding opportunities, and health-focused foundations can further their mission by providing funding for trail-related projects.

## Appendix. Interview Guide for Sunflower Trails Key Informant Interviews

This file is available for download as a Microsoft Word document.
