# Supplementary material for: Efforts of a Kansas Foundation to Increase Physical Activity and Improve Health by Funding Community Trails, 2012
Source: Prev Chronic Dis. 2014 Nov 26;11:E208. doi: 10.5888/pcd11.140356 (PMC4248789; doi:10.5888/pcd11.140356)
Supplement: Supplementary file 1 [file 14_0356_01.doc]

**Interview Guide for Sunflower Trails Key Informant Interviews**

Opening Script:

Thank you for the opportunity to interview you about your experience with funding from the Sunflower Trails program. We appreciate you volunteering your time to answer a few questions for our research evaluation. This interview will last approximately 20 to 30 minutes. You will be asked a few questions about your funded trail project.

This interview will be audio-taped and transcribed. No names or communities will be used in the transcriptions and only job titles (such as manager, supervisor, etc.) or project characteristics will be used when we compile the data. At the end of the study, recording files will be destroyed.

After we have completed all our scheduled interviews, we will compile the results and write a report. We will share this information with other researchers, policymakers, community organizations, and you. Again, your name will not be linked to any of the publicly available results.

Your participation in this interview is voluntary. If you would like to stop the interview at any time, you may do so without any negative consequences to you. Do you have any questions before we get started?

We will now turn on the recording device.

Interview Questions:

1. *Collaborative process of building the trail*

First, we would like to ask you a few questions about preparing your Trails application for the Sunflower Foundation.

How did you learn about the grant funding available for building a trail?

Please describe the process of preparing your application, including who you worked with in developing your plan? [probe: (a), (b), (c), or (d), if needed]

*How did the idea of building a trail come about?*

*Can you describe how you collaborated with different types of community organizations (number and type)?*

*Can you explain how the trail was the work of interest groups (number and type) within the community?*

*OR*

*Can you explain how the trail was the work of a stand-alone organization?*

Who was the “champion” for the trail? (individual or organization or both)

After you received funding from the Sunflower Foundation, how did you work together (across organizations) to build the trail? [probe: (a), (b), (c), if needed]

*After submitting the application, did the collaboration change or increase during trail construction?*

*How did you “divide” the work?*

*Did additional community organizations come on-board (ie, Parks and Recreation Department, Public Works Department)?*

Please explain if the trail was more difficult, as expected, or easier to complete than planned.

Please explain how you handled any extra costs for the trail, if any.

1. *Integration of the trail within the community*

We’re also interested in knowing how the trail has been integrated into the community since its construction.

How was the trail announced, marketed, and/or advertised?

Can you tell us a little bit about who is in charge of or responsible for the trail now that it’s been built?

Are there any events, promotions, or programs associated with the trail? [probe: (a) if needed]

*Are these [events, promotions, programs] formal or informal? Who are they organized by? Are they infrequent or scheduled on a regular basis?*

1. *Impact of the trail*

The last major topic we’d like to explore with you is the impact the trail has had within the community [may have already touched on this a bit; if so, acknowledge that briefly].

Can you talk about how the trail is used or has made a difference to various groups? [probe (a), (b), (c), or (d), if needed]

*Have you done any formal or informal assessment for the trail? What have you learned? How often is this done? Any plans to continue/expand these assessments?*

*Can you please describe the individuals or groups that use the trail?*

*What methods have you used to track who is using the trail?*

*Please explain how the trail might be changing physical activity levels.*

Are there any future plans for THIS trail in the community?

Has the trail led to OTHER trail-related/relevant projects? If so, please describe them.

1. *Do you have any final questions for me or any other information you would like to share?*

Closing Script:

Thank you so much for taking the time to do this interview! We really appreciate your input, and the information you have shared today will be incredibly helpful to us. In the next couple of weeks, we will provide you with a transcribed copy of this interview for you to verify. A full report of this evaluation will be placed on the Sunflower Foundation website. Have a great day!
